# Intravascular Ultrasound Guidance Improves the Long-term Prognosis in Patients with Unprotected Left Main Coronary Artery Disease Undergoing Percutaneous Coronary Intervention

**DOI:** 10.1038/s41598-017-02649-5

**Published:** 2017-05-24

**Authors:** Jian Tian, Changdong Guan, Wenyao Wang, Kuo Zhang, Jue Chen, Yongjian Wu, Hongbing Yan, Yanyan Zhao, Shubin Qiao, Yuejin Yang, Gary S. Mintz, Bo Xu, Yida Tang

**Affiliations:** 1 Department of Cardiology, Fu Wai Hospital, National Center for Cardiovascular Diseases, Chinese Academy of Medical Sciences and Peking Union Medical College, Beijing, China; 2 Catheterization Laboratories, Fu Wai Hospital, National Center for Cardiovascular Diseases, Chinese Academy of Medical Sciences and Peking Union Medical College, Beijing, China; 3 Department of Biostatistics, Fu Wai Hospital, National Center for Cardiovascular Diseases, Chinese Academy of Medical Sciences and Peking Union Medical College, Beijing, China; 4 The Cardiovascular Research Foundation, New York, NY USA

## Abstract

This study compared the long term outcomes in patients with unprotected left main coronary artery (LMCA) disease who underwent stenting under the guidance of intravascular ultrasound (IVUS) or conventional angiography at a large single center. The primary outcome was the composite of all-cause death and myocardial infarction (MI) at 3 years. Target vessel revascularization (TVR) at 3 years was one of the secondary outcomes. Between January 2004 and December 2011, a total of 1,899 patients who underwent IVUS-guided (n = 713, 37.5%) or conventional angiography-guided (n = 1186, 62.5%) stenting were included. At 3 years, the unadjusted primary outcome trended lower in the IVUS-guided group versus the angiography-guided (6.9% vs. 8.4%, p = 0.22) although the TVR was similar between two groups (6.0% vs. 6.0%, p = 0.97). However, after adjustment for differences in baseline risk factors, IVUS-guidance was associated with significantly lower incidence of the composite of all-cause death and MI (hazard ratio [HR]: 0.65; 95% confidence interval [CI]: 0.50 to 0.84; p = 0.001), although there was still no significant difference in TVR between the two groups (HR: 1.09; 95% CI: 0.84 to 1.42; p = 0.53). IVUS guidance has benefits in improving the long-term prognosis for unprotected LMCA stenting.

## Introduction

Percutaneous coronary intervention (PCI) for unprotected left main coronary artery (LMCA) disease is considered challenging because unprotected LMCA disease is associated with a relatively high risk of restenosis, myocardial infarction (MI), and mortality^1^. However, in selected patients PCI may be feasible and may provide equivalent results to coronary artery bypass grafting (CABG)^2^. Furthermore, improving long-term outcomes of PCI for unprotected LMCA disease may be facilitated by accurate assessment of lumen area and vessel size and plaque composition and distribution; however, angiography has many limitations in assessing LMCA size and plaque composition including the frequent lack of normal reference segments necessary for stent sizing^3^. Recent meta-analyses have demonstrated that intravascular ultrasound (IVUS) improved on the limitations of angiography; and IVUS-guided PCI is associated with lower risk of death, MI, target lesion revascularization (TLR), and stent thrombosis after drug-eluting stent (DES) implantation^4, 5^. In addition and in the setting of LMCA disease, the beneficial effects of IVUS-guidance on clinical outcomes have been shown in the MAIN-COMPARE registry which enrolled 975 patients (756 IVUS guidance vs. 219 conventional angiography)^6^. Since then, there have been only a few large clinical studies concentrating on IVUS’s impact on unprotected LMCA PCI^7–11^. In this current study we sought to substantiate the safety and efficacy of IVUS-guided stent implantation on the long-term prognosis of patients who underwent unprotected LMCA stenting.

## Method

### Population

Consecutive patients with unprotected LMCA disease who underwent elective PCI at Fu Wai Hospital (Beijing, China) between January 2004 and December 2011 were included in the current analysis. Patients with acute MI within 72 hours, treatment without stent implantation, bleeding history within the prior 3 months, cancer or other severe comorbidity affecting the life expectancy and known allergy to heparin, aspirin, or clopidogrel were excluded. This study was approved by the institutional review board central committee at Fuwai Hospital, NCCD of

China. All procedures were performed with standard interventional techniques following guidelines at that time. All patients enrolled in the study provided informed consent for angiography, PCI, IVUS usage if necessary and blood extraction before the angiography.

### Procedures

Use of IVUS was determined by each operator, and IVUS images were obtained using manual transducer pullback (40 MHz IVUS catheter, Boston Scientific, Minneapolis, Minnesota, USA) with commercially available imaging systems (Boston Scientific). IVUS was used both prior to and after stenting. IVUS criteria of stent optimization were as follows: 1) complete stent-to-vessel wall apposition; 2) adequate stent expansion (i.e., in-stent lumen cross-sectional area [CSA] of the target lesion ≥90% of the distal reference); and 3) full lesion coverage^12^. Anti-platelet therapy and periprocedural anticoagulation followed standard regimens. Before the procedure, patients received loading doses of aspirin (300 mg) and clopidogrel (300 mg), unless they had previously received regular anti-platelet medications. After the procedure, patients were maintained on aspirin (100 mg once daily) and clopidogrel (75 mg once daily) for at least 1 year after DES and for at least 6 months after bare metal stent placement, with longer treatment with clopidogrel at each operator’s discretion.

### Outcomes and Definitions

Post-procedure clinical assessment was performed at 30 days, 6 months, 1 year, 2 years, and 3 years either by clinic visits or telephone interviews. The primary outcome was the composite of all-cause death and myocardial infarction (MI) at 3 years. -All-cause death, cardiac death, MI, Q-wave MI, target vessel related myocardial infarction (TV-MI), definite/probable stent thrombosis (ST), target vessel revascularization (TVR), any revascularization and target lesion revascularization (TLR) were considered to be the secondary outcomes of the study. MI was defined as creatine kinase concentration of >2× the upper limit of normal. Definite or probable stent thrombosis was defined according to the recommendations of the Academic Research Consortium^13^ and TVR as any revascularization within the entire major coronary vessels proximal or distal to a target lesion including upstream and downstream side branches and the target lesion itself.

### Statistical Analysis

Differences were compared using the Student’s t-test (for normal data) or Mann–Whitney U-test (for non-normally distributed variables) for continuous variables as appropriate and the χ^2^ test or Fisher exact test for categorical variables. All reported P values were 2-sided, and P < 0.05 were considered to indicate statistical significance. The probability of IVUS guidance or not (propensity score [PS]) being conditioned by observed baseline characteristics was estimated by multiple logistic regression. A full nonparsimonious model was developed, which included all the variables shown in Supplementary Table 1. PS matching and trimmed inverse-probability-of-treatment weighting (IPW) were used to reduce the treatment selection bias and potential confounding factors in this study. Patients were matched (a 1:1 match) on the logit of the PS using a caliper of width equal to 0.1 standard deviations of the logit of PS. For trimmed-IPW, the weights for patients undergoing IVUS guidance were the inverse of propensity score; and weights for patients receiving angiographic guidance were the inverse of 1-propensity score. Model discrimination was assessed with c-statistics, and baseline characteristics of patients after PS match and adjustment with trimmed-IPW were presented as standardized difference (Supplementary Tables 2 and 3). SAS 9.1 (SAS Institute, Cary, North Carolina, USA) was used for statistical analysis.

## Results

### Patient Characteristics

**Figure 1:**
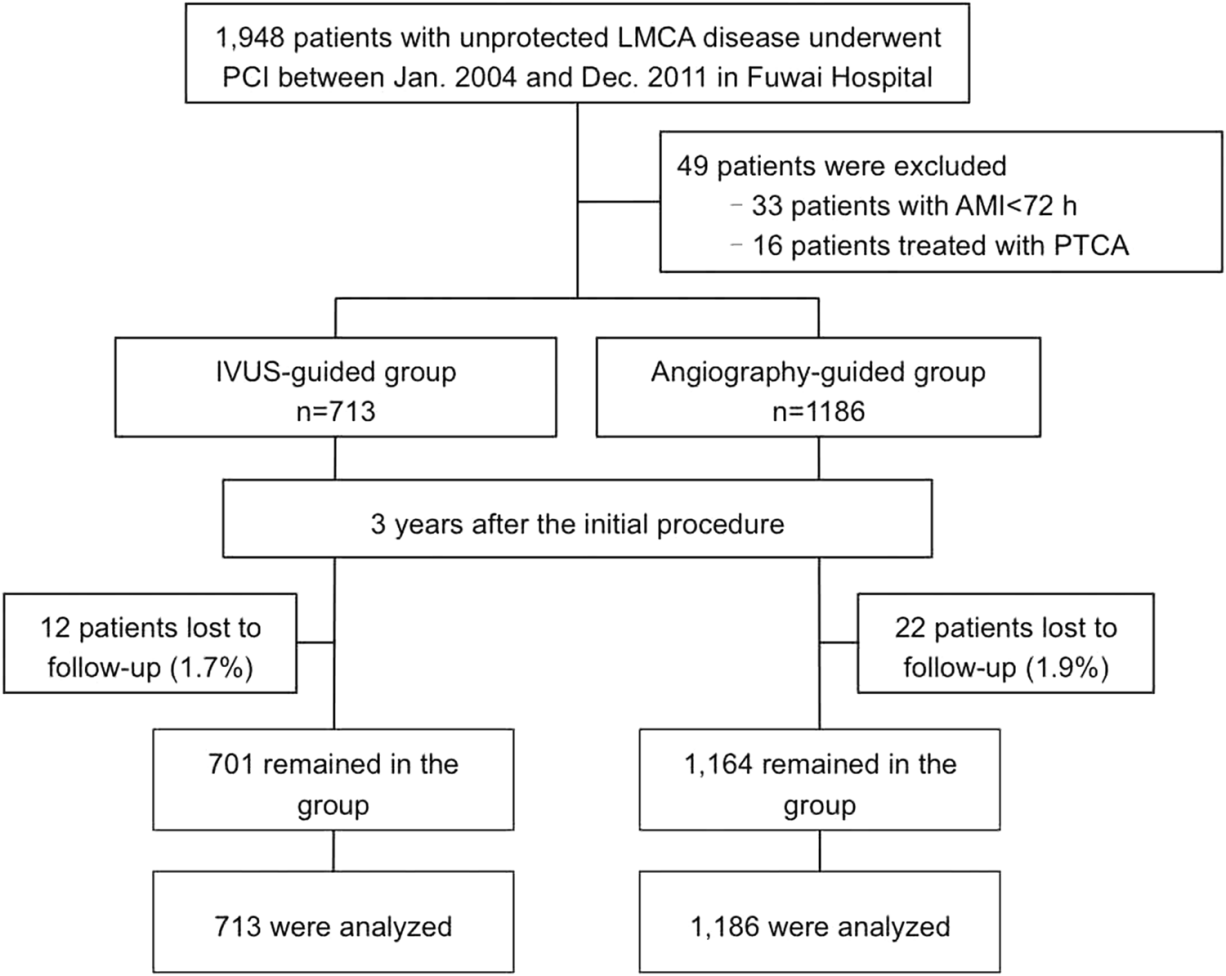
Patient Flowchart. AMI = acute myocardial infarction; IVUS = intravascular ultrasound; LMCA = left main coronary artery; PCI = percutaneous coronary intervention; PTCA = percutaneous transluminal coronary angioplasty.

A total of 1,899 patients were included in this analysis: 713 (37.5%) underwent IVUS-guided stenting, and 1186 (62.5%) underwent conventional angiography-guided stenting. Overall, 98.2% of patients completed 3-year follow-up (Fig. 1). The unadjusted baseline clinical characteristics of the two groups have been listed in Table 1. All clinical characteristics were similar comparing the IVUS vs. angiography guidance groups except that there were more current smokers (35.9% vs. 26.6%, p < 0.01) and more patients with isolated LM disease (8.3% vs. 5.6%, p = 0.02) in the IVUS guidance group.

**Table 1:** Baseline Patient and Lesion Characteristics.

Variable	IVUS guidance (n = 713)	Angiography guidance (n = 1186)	p Value
Age	59.6 ± 10.9	60.0 ± 10.2	0.45
Male	576 (80.8)	920 (77.6)	0.10
BMI, kg/m^2^	25.6 ± 3.0	25.8 ± 3.3	0.09
Hypertension	400 (56.1)	654 (55.1)	0.68
Hyperlipidemia	387 (54.3)	597 (50.3)	0.10
Diabetes mellitus	173 (24.3)	314 (26.5)	0.28
Family history of CAD	111 (15.6)	172 (14.5)	0.53
Previous MI	163 (22.9)	293 (24.7)	0.36
Previous PCI	165 (23.1)	270 (22.8)	0.85
Previous stroke	50 (7.0)	85 (7.2)	0.90
Peripheral vascular disease	45 (6.3)	56 (4.7)	0.14
Smoking history			<0.01
Current smoker	256 (35.9)	316 (26.6)	<0.01
Ex-smoker	128 (18.0)	230 (19.4)	0.44
Non-smoker	329 (46.1)	640 (54.0)	<0.01
Clinical presentation			0.47
Stable angina	236 (33.1)	401 (33.8)	0.75
Unstable angina	452 (63.4)	755 (63.7)	0.91
Silent ischemia	25 (3.5)	30 (2.5)	0.22
Creatinine, μmol/L	80.4 ± 16.6	81.3 ± 18.9	0.32
Creatinine clearance rate, ml/min	89.7 ± 28.8	88.6 ± 27.4	0.40
LVEF,%	63.2 ± 6.8	62.9 ± 7.4	0.32
Baseline SYNTAX score	23.7 ± 7.1	24.1 ± 7.1	0.21
Number of target lesion per patient	1.70 ± 0.76	1.68 ± 0.80	0.58
Angiographic findings			0.09
Isolated LM	59 (8.3)	66 (5.6)	0.02
LM + 1 vessel	141 (19.8)	240 (20.2)	0.81
LM + 2 vessel	258 (36.2)	416 (35.1)	0.62
LM + 3 vessel	255 (35.8)	464 (39.1)	0.14
LM lesion type			0.24
De novo	691 (96.9)	1160 (97.8)	
Restenosis	22 (3.1)	26 (2.2)	
LM lesion location			0.65
Ostium	81 (11.4)	148 (12.5)	0.47
Shaft	45 (6.3)	82 (6.9)	0.61
Distal bifurcation	587 (82.3)	956 (80.6)	0.35

Values are mean ± SD or n (%).

BMI = body mass index; CABG = coronary artery bypass graft; CAD = coronary artery disease; IVUS = intravascular ultrasound; LM = left main; LVEF = left ventricular ejection fraction; MI = myocardial infarction; PCI = percutaneous coronary intervention; SYNTAX = synergy between PCI with TAXUS and cardiac surgery.

### PCI Procedure Details

PCI details have been listed in Table 2. Although patients in the IVUS-guidance group had similar pre-procedure SYNTAX scores (23.7 ± 7.1 vs. 24.1 ± 7.1, p = 0.21) and a similar prevalence of LMCA bifurcation lesions (82.3% vs. 80.6%, p = 0.35), these patients had a longer PCI duration time (67.9 ± 37.5 min vs. 44.6 ± 30.7 min, p < 0.01), lesions that were treated with shorter stents (length 26.6 ± 15.7 mm vs. 29.3 ± 17.6 mm, p < 0.01), and lesion in which larger stents were implanted (diameter 3.54 ± 0.51 mm vs. 3.39 ± 0.48 mm, p < 0.01) due to IVUS-measured shorter lesion length and larger vessel size.

**Table 2:** Procedural Characteristics.

Variable	IVUS guidance (n = 713)	Angiography guidance (n = 1186)	p Value
Transradial approach	453 (63.5)	794 (66.9)	0.13
Total lesion length, mm	21.7 ± 15.3	24.3 ± 17.0	<0.01
Stents per patient	2.20 ± 1.10	2.22 ± 1.18	0.74
Stents diameter, mm	3.54 ± 0.51	3.39 ± 0.48	<0.01
Total stent length per patient, mm	26.7 ± 15.7	29.3 ± 17.6	<0.01
Type of stent			0.10
1^st^ generation DES	496 (69.6)	802 (67.5)	0.38
2^nd^ generation DES	208 (29.1)	349 (29.5)	0.91
BMS	9 (1.3)	35 (3.0)	0.02
LM bifurcation lesions	587 (82.3)	956 (80.6)	0.35
One-stent strategy	321 (54.7)	720 (75.3)	<0.01
Two-stent strategy	266 (45.3)	236 (24.7)	<0.01
Culotte	7 (2.6)	18 (7.6)	0.01
Crush	185 (69.5)	161 (68.2)	0.75
Kissing or V	39 (14.7)	18 (7.6)	0.01
T	35 (13.2)	39 (16.5)	0.29
Final kissing balloon inflation	399 (68.0)	395 (41.3)	<0.01
Post-dilation	551 (77.3)	638 (53.8)	<0.01
Maximum diameter of post-dilation balloon, mm	4.03 ± 0.44	3.88 ± 0.48	<0.01
Maximum pressure of the largest post-dilation balloon, atm	17.50 ± 3.84	16.93 ± 4.44	0.02
Procedural complications	19 (2.7)	44 (3.7)	0.21
PCI duration, min	67.9 ± 37.5	44.6 ± 30.7	<0.01
Residual SYNTAX score	3.65 ± 4.66	4.60 ± 5.59	<0.01
Procedural success	709 (99.4)	1181 (99.6)	0.74

Values are mean ± SD or n (%).

BMS = bare metal stent; DES = drug-eluting stent; other abbreviations as in Table 1.

Post-dilation was more frequently used in the IVUS-guided group (77.3% vs. 53.8%, p < 0.01) with bigger post-dilation balloons (4.03 ± 0.44 mm vs. 3.88 ± 0.48 mm, p < 0.01) and higher inflation pressures (17.5 ± 3.84 atm vs. 16.9 ± 4.44 atm, p = 0.02). For LM bifurcation lesions, there were more final kissing balloon inflations (68.0% vs. 41.3%, p < 0.01) and more frequent use of a two-stent technique (45.3% vs. 24.7%, p < 0.01) in the IVUS guidance group. The post-procedure Residual SYNTAX Score (3.65 ± 4.66 vs. 4.60 ± 5.59, p < 0.01) in the IVUS guidance group was significantly lower than in the angiography-guided group.

### Long-term Clinical Outcomes

**Figure 2:**
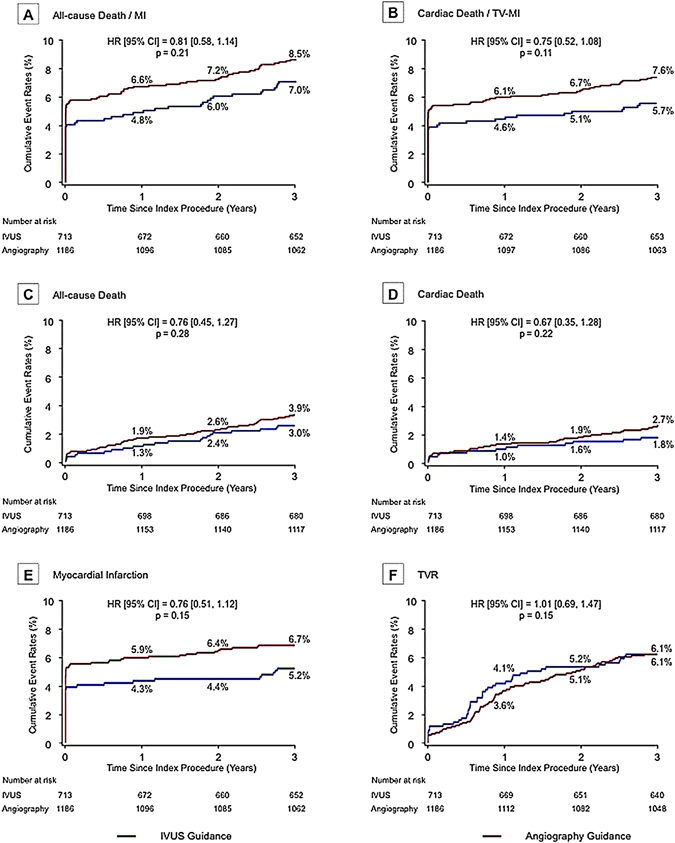
Unadjusted Kaplan-Meier Curves of 3-Year Outcomes. The HRs were reported for patients with IVUS guided versus those without IVUS guided. CI = confidence interval; HR = hazard ratio; TV-MI = target vessel myocardial infarction; TVR = target vessel revascularization; other abbreviations as in Fig. 1.

**Figure 3:**
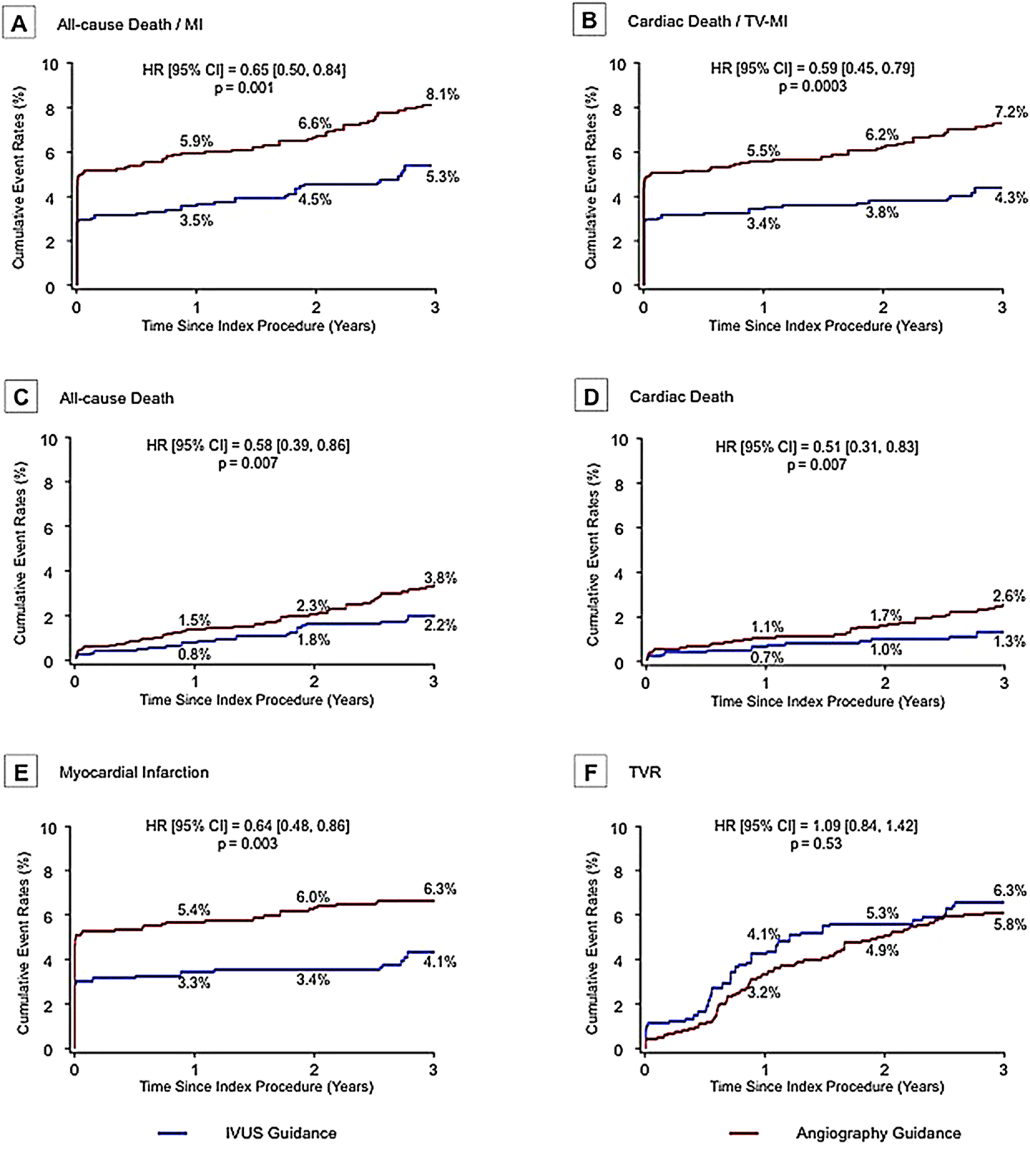
Adjusted Kaplan-Meier Curves of 3-year Outcomes. Trimmed inverse probability weighted Cox proportional-hazards regression was used with adjustment for all patient-level variables in Supplementary Table 1. The HRs were reported for patients with IVUS guided versus those without IVUS guided. Abbreviations as in Figs 1 and 2.

The observed (unadjusted) clinical outcomes through 3 years have been presented in Fig. 2 and Table 3. There was a trend toward lower rates of death (2.9% vs. 3.9%, p = 0.29) and MI (5.2% vs. 6.8%, p = 0.16) in the IVUS guidance group, but without significant difference. However, after adjustment of baseline covariates with trimmed-IPW, the trend was prominent between two groups. The trimmed-IPW model indicated good predictive value (C-statistic 0.78); and 99% of all patients (n = 1880) could be entered into the final analysis. The adjusted Cox regression analysis showed that the incidence of the primary outcome (composite of all-cause death and MI) was significantly lower in the IVUS-guidance group compared to the angiography-guidance group (hazard ratio [HR]: 0.65, 95% confidence interval [CI]: 0.50 to 0.84, p = 0.001). There were also significantly lower risks of 3-year all-cause death (HR: 0.58, 95% CI: 0.39 to 0.86; p = 0.007), cardiac death (HR: 0.51, 95% CI: 0.31 to 0.83, p = 0.007), and MI (HR: 0.64, 95% CI: 0.48 to 0.86, p = 0.003), but not the risk of TVR (HR: 1.09, 95% CI: 0.84 to 1.42, p = 0.53). The Kaplan-Meier curves for MI events (IVUS-guided vs. angiography-guided) started separating early and continued to separate (Fig. 3).

**Table 3:** Clinical Outcomes Through 3 Years.

	IVUS guidance (n = 713)	Angiography guidance (n = 1186)	Unadjusted		PS match (n = 542 pairs)		Adjusted with Trimmed-IPW	
			Hazard ratio (95%CI)	p Value	Hazard ratio (95%CI)	p Value	Hazard ratio (95%CI)	p Value
**30 days**								
All-cause death	3 (0.4)	10 (0.8)	0.50 (0.14, 1.81)	0.29	0.20 (0.02, 1.71)	0.14	0.33 (0.10, 1.10)	0.07
Cardiac death	3 (0.4)	8 (0.7)	0.62 (0.17, 2.35)	0.48	0.25 (0.03, 2.24)	0.22	0.37 (0.11, 1.24)	0.11
Myocardial infarction	28 (3.9)	66 (5.6)	0.71 (0.45, 1.10)	0.71	0.50 (0.28, 0.90)	0.02	0.57 (0.40, 0.80)	0.001
Q-wave MI	3 (0.4)	15 (1.3)	0.33 (0.10, 1.15)	0.08	0.13 (0.02, 1.00)	0.05	0.20 (0.07, 0.62)	0.005
TV-MI	28 (3.9)	65 (5.5)	0.72 (0.46, 1.11)	0.14	0.50 (0.28, 0.90)	0.02	0.57 (0.41, 0.81)	0.001
All-cause death/MI	29 (4.1)	69 (5.8)	0.70 (0.45, 1.08)	0.10	0.49 (0.27, 0.87)	0.01	0.57 (0.41, 0.79)	0.0009
Cardiac death/TV-MI	29 (4.1)	67 (5.6)	0.72 (0.47, 1.11)	0.14	0.49 (0.27, 0.87)	0.01	0.58 (0.42, 0.82)	0.002
Definite/probable ST	3 (0.4)	7 (0.6)	0.71 (0.18, 2.76)	0.62	0.67 (0.11, 3.99)	0.66	0.68 (0.23, 1.97)	0.48
Any revascularization	8 (1.1)	10 (0.8)	1.33 (0.53, 3.37)	0.55	1.00 (0.25, 4.00)	1.00	1.81 (0.87, 3.79)	0.12
TVR	8 (1.1)	7 (0.6)	1.90 (0.69, 5.24)	0.21	1.00 (0.25, 4.00)	1.00	2.79 (1.19, 6.56)	0.02
TLR	6 (0.8)	4 (0.3)	2.50 (0.70, 8.84)	0.16	1.00 (0.20, 4.96)	1.00	4.77 (1.55, 14.6)	0.006
**1 year**								
All-cause death	9 (1.3)	23 (1.9)	0.65 (0.30, 1.40)	0.27	0.25 (0.07, 0.89)	0.03	0.54 (0.29, 1.02)	0.06
Cardiac death	7 (1.0)	16 (1.3)	0.73 (0.30, 1.76)	0.48	0.43 (0.11, 1.66)	0.22	0.61 (0.30, 1.27)	0.19
Myocardial infarction	31 (4.3)	71 (6.0)	0.72 (0.48, 1.10)	0.13	0.51 (0.30, 0.89)	0.02	0.60 (0.43, 0.83)	0.002
Q-wave MI	6 (0.8)	18 (1.5)	0.55 (0.22, 1.39)	0.21	0.33 (0.09, 1.23)	0.10	0.49 (0.24, 1.01)	0.05
TV-MI	31 (4.3)	69 (5.8)	0.75 (0.49, 1.14)	0.17	0.53 (0.30, 0.92)	0.02	0.61 (0.44, 0.85)	0.003
All-cause death/MI	34 (4.8)	80 (6.7)	0.73 (0.49, 1.08)	0.11	0.45 (0.26, 0.78)	0.004	0.59 (0.44, 0.81)	0.0009
Cardiac death/TV-MI	33 (4.6)	74 (6.2)	0.74 (0.49, 1.11)	0.15	0.49 (0.28, 0.84)	0.01	0.61 (0.44, 0.84)	0.002
Definite/probable ST	7 (1.0)	11 (0.9)	1.06 (0.41, 2.73)	0.91	1.67 (0.40, 6.97)	0.48	1.11 (0.52, 2.34)	0.79
Any revascularization	39 (5.5)	68 (5.7)	0.95 (0.64, 1.41)	0.80	0.93 (0.56, 1.56)	0.79	1.13 (0.85, 1.50)	0.39
TVR	29 (4.1)	42 (3.5)	1.15 (0.72, 1.85)	0.56	1.18 (0.62, 2.25)	0.62	1.29 (0.92, 1.83)	0.14
TLR	15 (2.1)	27 (2.3)	1.23 (0.66, 2.30)	0.52	1.22 (0.51, 2.95)	0.66	1.35 (0.85, 2.15)	0.21
**3 years**								
All-cause death	21 (2.9)	46 (3.9)	0.76 (0.45, 1.27)	0.29	0.42 (0.21, 0.86)	0.02	0.58 (0.39, 0.86)	0.007
Cardiac death	13 (1.8)	32 (2.7)	0.67 (0.35, 1.28)	0.23	0.50 (0.21, 1.17)	0.11	0.51 (0.31, 0.83)	0.007
Myocardial infarction	37 (5.2)	81 (6.8)	0.76 (0.51, 1.12)	0.16	0.57 (0.35, 0.94)	0.03	0.64 (0.48, 0.86)	0.003
Q-wave MI	12 (1.7)	30 (2.5)	0.66 (0.34, 1.29)	0.22	0.50 (0.21, 1.17)	0.11	0.61 (0.38, 1.00)	0.05
TV-MI	36 (5.0)	79 (6.7)	0.76 (0.51, 1.12)	0.16	0.56 (0.34, 0.94)	0.03	0.63 (0.47, 0.84)	0.002
All-cause death/MI	49 (6.9)	100 (8.4)	0.81 (0.58, 1.14)	0.22	0.56 (0.36, 0.87)	0.01	0.65 (0.50, 0.84)	0.001
Cardiac death/TV-MI	41 (5.8)	91 (7.7)	0.75 (0.52, 1.08)	0.12	0.54 (0.34, 0.87)	0.01	0.59 (0.45, 0.79)	0.0003
Definite/probable ST	10 (1.4)	20 (1.7)	0.83 (0.39, 1.77)	0.63	0.88 (0.32, 2.41)	0.80	0.77 (0.44, 1.35)	0.36
Any revascularization	60 (8.4)	114 (9.6)	0.87 (0.64, 1.19)	0.38	0.86 (0.57, 1.29)	0.46	0.98 (0.79, 1.23)	0.89
TVR	43 (6.0)	71 (6.0)	1.01 (0.69, 1.47)	0.97	0.94 (0.57, 1.54)	0.80	1.09 (0.84, 1.42)	0.53
TLR	22 (3.1)	39 (3.3)	0.94 (0.56, 1.58)	0.81	0.83 (0.42, 1.65)	0.60	1.09 (0.76, 1.58)	0.64

Values are mean ± SD or n (%).

CI = confidence interval; IPW = inverse probability weight; ST = stent thrombosis; TV-MI = target vessel myocardial infarction; TVR = target vessel revascularization; TLR = target lesion revascularization; other abbreviations as in Table 1.

After performing PS matching in the entire population, a total of 542 matched pairs of patients were created (C-statistic 0.77). The results were consistent with trimmed-IPW. The primary outcome was significantly lower in the IVUS-guidance group (HR: 0.56, 95% CI: 0.36 to 0.87; p = 0.01), but there was no significant difference in terms of TVR (HR: 0.94, 95% CI: 0.57 to 1.54; p = 0.80) (Table 3 and Supplementary Figure 1).

## Discussion

In this study we found that IVUS-guided stenting for unprotected LMCA disease reduced the primary safety outcome, but not the risk of TVR compared with angiography-guided stenting. The advantage of MI reduction seemed to be more obvious in the early stage of follow-up, suggesting a reduction in early stent thrombosis^14^; however, the curves continued to separate indicating an ongoing benefit to IVUS guidance.

These results were compatible to other previous studies such as the MAIN-COMPARE study^6^. In the MAIN-COMPARE study the analysis of 201 propensity-matched pairs of patients showed that the 3-year incidence of total mortality was lower in patients undergoing IVUS-guided stenting compared with angiography-guided stenting (4.7% vs. 16%; p < 0.05), but not the incidence of MI or TLR. Gao *et al*. showed that after propensity-score matching, IVUS-guided stenting was associated with reduced 1-year MACE, mainly driven by a decrease in cardiac death and TVR^9^. De La Torre Hernandez *et al*. reported a better survival free of cardiac death, MI, and TLR at 3 years in the IVUS-guided group vs. the angiography-guided group with a lower incidence of definite and probable ST^8^. More importantly, the sole randomized clinical trial specifically addressing patients with LMCA disease, albeit in only 123 patients, showed that IVUS guidance was associated with a reduction in 2-year major adverse cardiac events from 29.3% to 13.1% (p = 0.031) as well as a reduction in TLR from 24.0% to 9.1% (p = 0.045)^7^. Compared to the published studies, the 1899 patient cohort in this current study was the largest unprotected LMCA stenting cohort reported so far. The baseline characteristics were well balanced even before the trimmed IPW adjustment, which guaranteed that its results could provide reliable evidence. At Fuwai Hospital, the PCI for unprotected LMCA can only be performed by experienced operators whose skills have been well maintained to insure sustained PCI results and avoid potential operator bias.

There were several explanations for the benefits of IVUS-guidance. First, IVUS guidance provided a more accurate assessment of lesion severity^15^ and lesion length^16^. Suh *et al*.^17^ found that stent length was an independent predictor of stent thrombosis. Second, LMCA disease frequently does not have recognizable reference segments^18^; this impacts both assessment of lesion severity and also PCI strategy including stent size and length selection. Third, the angiographic classification of distal LMCA bifurcation lesions is frequently misleading; IVUS assessment provides a more accurate assessment of LMCA disease extension into the proximal LAD and/or LCX. In Oviedo *et al*.’s retrospective study^19^, IVUS analysis showed that bifurcation disease was diffuse rather than focal; and continuous plaque from the LMCA to the LAD and/or LCX was seen at a much higher rate than with angiography. Han *et al*.^20^ found that the percentage of necrotic core and dense calcium at the LMCA bifurcations was significantly higher than in proximal segments. Thus, IVUS guidance may be helpful in choosing a more appropriate PCI strategy and in getting better acute post-procedure results; this then translates into better long-term outcomes. In our study, stent diameter was much larger and more post-dilation (77.3% vs. 53.8%, p < 0.01) was performed with larger post-dilation balloons (4.03 ± 0.44 mm vs. 3.88 ± 0.48 mm, p < 0.01) and higher inflation pressures (17.5 ± 3.84 atm vs. 16.9 ± 4.44 atm, p = 0.02) in the IVUS guidance group than in the angiography guidance group. Furthermore, for LM bifurcation lesions, there were more final kissing balloon inflations and more two-stent techniques used in the IVUS guidance group. In Chen *et al*.’s^21^ study and as compared with angiography, IVUS-guidance helped operators to optimize the acute results of two-stent techniques for unprotected LMCA; this was associated with improved 1-year clinical outcomes as well a reduction in overall unadjusted ST(1.2% vs. 6.9%, p < 0.01), definite ST (0.6% vs. 5.3%, p < 0.01), late ST (0.6% vs. 4.3%, p < 0.01), MI (4.6% vs. 8.9%, p = 0.038) and cardiac death (0.9% vs. 3.3%, p = 0.049). De La Torre Hernandez *et al*. reported that IVUS guidance was especially beneficial in patients with distal bifurcation lesions^8^. Our results also showed that the patients in the IVUS-guided group had much lower post-procedure residual SYNTAX Scores than conventional angiography-guided group (3.65 ± 4.66 vs. 4.60 ± 5.59, p < 0.01) even though their pre-procedure SYNTAX scores were similar (23.7 ± 7.1 vs. 24.1 ± 7.1, p = 0.21). More complete revascularization may be associated with a reduction in late events.

### Study Limitations

Our study had several limitations, including use of single center data, operator’s discretion whether to use IVUS or rely on angiography alone, and the fact that the study was non-randomized and retrospective. Therefore, despite rigorous statistical adjustment, unmeasured confounders may have influenced the outcomes.

## Conclusion

IVUS-guided stenting had a benefit in reducing long-term mortality rates compared with angiography-guided stenting for unprotected LMCA stenosis. Further randomized controlled trials with larger sample size are needed to further address the real advantages of IVUS over angiography guidance in unprotected LMCA disease.

## Electronic supplementary material

Supplementary File
