# Supplementary material for: Intravascular Ultrasound Guidance Improves the Long-term Prognosis in Patients with Unprotected Left Main Coronary Artery Disease Undergoing Percutaneous Coronary Intervention
Source: Sci Rep. 2017 May 24;7:2377. doi: 10.1038/s41598-017-02649-5 (PMC5443793; doi:10.1038/s41598-017-02649-5)
Supplement: Supplementary file 1 — Supplementary File [file 41598_2017_2649_MOESM1_ESM.pdf]

Intravascular Ultrasound Guidance Improves the Long-term Prognosis in Patients with Unprotected Left Main Coronary Artery Disease Undergoing Percutaneous Coronary Intervention

Jian Tian, MD[1+], Changdong Guan, MSc[2+], Wenyao Wang, MD[1], Kuo Zhang, MD[1], Jue Chen, MD[1], Yongjian Wu, MD[1], Hongbing Yan, MD[1], Yanyan Zhao, BS[3], Shubin Qiao, MD[1], Yuejin Yang, MD[1], Gary S. Mintz, MD[4], Bo Xu, MBBS[2*], Yida Tang, MD, PhD[1*]

[1]Department of Cardiology, Fu Wai Hospital, National Center for Cardiovascular Diseases, Chinese Academy of Medical Sciences and Peking Union Medical College, Beijing, China; [2]Catheterization Laboratories, Fu Wai Hospital, National Center for Cardiovascular Diseases, Chinese Academy of Medical Sciences and Peking Union Medical College, Beijing, China; [3]Department of Biostatistics, Fu Wai Hospital, National Center for Cardiovascular Diseases, Chinese Academy of Medical Sciences and Peking Union Medical College, Beijing, China; [4]The Cardiovascular Research Foundation, New York, NY, USA

***Corresponding authors:**

Yi-Da Tang, MD, PhD, Professor and Chairman, Department of Internal Medicine， Deputy Director, Coronary Heart Disease Center, State Key

Laboratory of Cardiovascular Disease, Fuwai Hospital, National Center for Cardiovascular Diseases, Chinese Academy of Medical Sciences and Peking Union Medical College, No. 167 Beilishi Road, Beijing 100037, China. Tel: +86-10-88396171. Fax: +86-10-88396171. Email: tangyida@fuwaihospital.org

Bo Xu, MBBS, Catheterization Laboratories, Fu Wai Hospital, National Center for Cardiovascular Diseases, Chinese Academy of Medical Sciences and Peking Union Medical College, 167 Beilishi Road, Xicheng District, Beijing 100037, China. E-mail: bxu@citmd.com.

[+]These authors contributed equally to this work and should be considered co-first authors.

Supplementary Figure 1. Adjusted Kaplan-Meier Curves of 3-year Outcomes with PS Match.

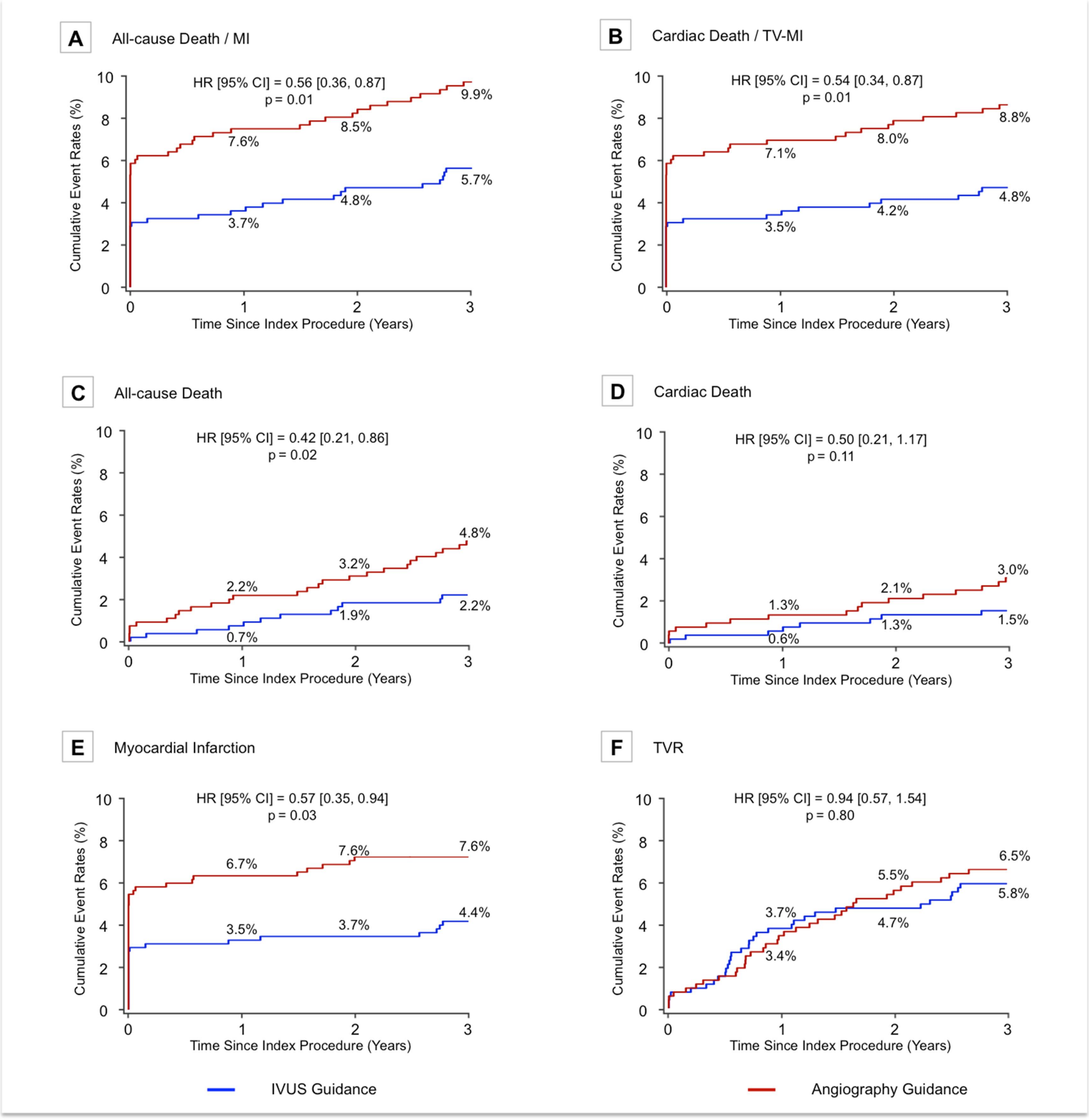

Supplementary Table 1. Candidate Covariates for Adjustment

Risk Factors		
1. Age	10. Hyperlipidemia	19. SYNTAX score II
2. Ejection fraction	11. Unstable angina	20. Residual SYNTAX score
3. Previous MI	12. De novo lesion	21. Duration of PCI
4. Previous PCI	13. Transradial approach	22. Number of target lesion
5. Previous CABG	14. LM lesion location	23. Length of target lesion
6. Diabetes mellitus	15. Type of stent	24. Number of stent
7. Hypertension	16. Dissection in target lesion	25. Total length of stents
8. Family history of CAD	17. Operation complication	26. Diameter of stents
9. Operators	18. SYNTAX score before PCI	27. Extent of diseased vessel

MI=myocardial infarction; PCI=percutaneous coronary intervention; CABG=coronary artery bypass grafting; CAD=coronary artery disease; LM=left main; SYNTAX=Synergy between Percutaneous Coronary Intervention with Taxus and Cardiac Surgery

Supplementary Table 2. Baseline Characteristics After trimmed-IPW Adjustment

**Variable**	**IVUS guidance (n=703)**	**Angiography guidance (n=1177)**	**p Value**
Age	59.8 ± 16.8	59.7 ± 13.1	0.88
Male	560 (79.6)	918(78.0)	0.26
BMI, kg/m^2	25.6 ± 4.7	25.9 ± 4.3	0.02
Hypertension	384 (54.6)	643 (54.6)	0.99
Hyperlipidemia	370 (52.6)	606 (51.5)	0.54
Diabetes mellitus	178 (25.3)	300 (25.5)	0.91
Family history of CAD	108 (15.4)	175 (14.9)	0.68
Previous MI	169 (24.1)	282 (24.0)	0.92
Previous PCI	157 (22.3)	265 (22.5)	0.87
Previous stroke	43 (6.1)	89 (7.6)	0.08
Peripheral vascular disease	42 (6.0)	54 (4.6)	0.06
Smoking history			<0.01
Current smoker	247 (35.2)	321 (27.3)	
Ex-smoker	128 (18.2)	230 (19.5)	
Non-smoker	328 (46.6)	626 (53.2)	
Clinical presentation			0.82
Stable angina	229 (32.6)	397 (33.5)	
Unstable angina	452 (64.4)	750 (63.7)	
Silent ischemia	22 (3.0)	30 (2.8)	
Creatinine, µmol/L	80.3±26.4	80.8±23.6	0.49
Creatinine clearance rate, ml/min	89.3±44.1	89.9±34.6	0.68
LVEF,%	62.6±11.3	62.9±9.0	0.60
Baseline SYNTAX score	23.9±11.6	24.0±8.9	0.84
Number of target lesion per patient	1.68±1.18	1.80±1.02	0.58
Angiographic findings			0.81
Isolated LM	51 (6.8)	66 (6.3)	
LM+1 vessel	140 (20.0)	238 (20.2)	
LM+2 vessel	257 (36.5)	416 (35.5)	
LM+3 vessel	255 (36.7)	457 (38.0)	

LM lesion type			0.80
De novo	686 (97.6)	1150 (97.7)	
Restenosis	17 (2.4)	27 (2.3)	
LM lesion location			0.97
Ostium	81 (12.5)	144 (12.2)	
Shaft	45 (6.5)	79 (6.7)	
Distal bifurcation	577 (81.0)	954 (81.1)	
Transradial approach	467 (66.4)	784 (66.6)	0.13
Total lesion length, mm	22.7±26.2	23.3±20.6	0.44
Procedural complications	19 (2.7)	38 (3.3)	0.95
PCI duration, min	56.7±47.3	52.6±45.2	0.01
Residual SYNTAX score	4.17±8.27	2.24±6.70	0.78
Procedural success	699 (99.5)	1173 (99.7)	0.49

Supplementary Table 3. Baseline Characteristics After PS Match

Variable	IVUS guidance (n=542)	Angiography guidance (n=542)	p Value
Age	59.3 ± 10.6	59.3 ± 10.7	0.98
Male	433 (79.9)	432(79.7)	0.94
BMI, kg/m²	25.6 ± 2.8	25.8 ± 3.5	0.28
Hypertension	302 (55.7)	289 (53.3)	0.42
Hyperlipidemia	283 (52.2)	275 (50.7)	0.63
Diabetes mellitus	128 (23.6)	120 (22.1)	0.58
Family history of CAD	87 (16.1)	79 (14.6)	0.50
Previous MI	133 (24.5)	123 (22.7)	0.47
Previous PCI	117 (21.6)	119 (22.0)	0.88
Previous stroke	35 (6.5)	37 (6.8)	0.81
Peripheral vascular disease	32 (5.9)	20 (3.7)	0.09
Smoking history			<0.01
Current smoker	200 (36.9)	136 (25.1)	
Ex-smoker	97 (17.9)	110 (20.3)	
Non-smoker	245 (45.2)	296 (54.6)	
Clinical presentation			0.77
Stable angina	178 (32.8)	191 (35.2)	
Unstable angina	345 (63.7)	336 (62.0)	
Silent ischemia	19 (3.5)	15 (2.8)	
Creatinine, μmol/L	79.8±16.5	81.2±20.1	0.19
Creatinine clearance rate, ml/min	90.3±25.8	89.9±26.9	0.81
LVEF,%	62.9±7.0	62.6±7.2	0.39
Baseline SYNTAX score	23.9±7.1	23.6±7.3	0.50
Number of target lesion per patient	1.69±0.76	1.66±0.82	0.50
Angiographic findings			0.51
Isolated LM	35 (6.5)	42 (7.7)	
LM+1 vessel	114 (21.0)	121 (22.3)	
LM+2 vessel	195 (36.0)	197 (36.3)	
LM+3 vessel	198 (36.5)	182 (33.6)	

LM lesion type			0.68
De novo	527 (97.2)	529 (97.6)	
Restenosis	15 (2.8)	13 (2.4)	
LM lesion location			0.87
Ostium	68 (12.5)	66 (12.2)	
Shaft	32 (5.9)	39 (7.2)	
Distal bifurcation	442 (81.5)	437 (80.6)	
Transradial approach	356 (65.7)	359 (66.2)	0.84
Total lesion length, mm	22.5±16.1	22.5±15.6	0.99
Procedural complications	13 (2.4)	20 (3.7)	0.22
PCI duration, min	57.8±26.9	55.9±37.7	0.21
Residual SYNTAX score	3.95±4.91	4.01±5.28	0.84
Procedural success	538 (99.3)	538 (99.3)	1.00

Supplementary Table 4. Baseline Characteristics of Patients After Adjustment With Trimmed Inverse Probability Weight

Variable	Standardized Difference Without Adjustment	Standardized Difference With IPW Adjustment
Female	0.077	0.037
Age	0.041	0.005
BMI	0.073	0.075
LVEF	0.038	0.017
Previous MI	0.046	0.003
Previous PCI	0.003	0.005
Hypertension	0.022	0.000
Diabetes	0.053	0.004
Hyperlipidemia	0.082	0.020
Family history of CAD	0.031	0.014
Previous Stroke	0.001	0.059
Peripheral vascular disease	0.072	0.063
Smoking history		
None-smoker	0.153	0.133
Current-smoker	0.199	0.171
Ex-smoker	0.039	0.033
Clinical presentation		
Stable angina	0.012	0.019
Unstable angina	0.006	0.015

Variable	Standardized Difference Without Adjustment	Standardized Difference With IPW Adjustment
Silent ischemia	0.051	0.010
Transfemoral approach	0.071	0.004
PCI duration	0.719	0.088
Angiographic findings		
Isolated LM	0.109	0.019
LM+1 vessel	0.006	0.006
LM+2 vessel	0.020	0.021
LM+3 vessel	0.073	0.026
Restenosis	0.063	0.008
Total lesion length	0.159	0.025
LM lesion location		
Ostium	0.027	0.007
Shaft	0.023	0.005
Distal bifurcation	0.037	0.002
Complication	0.068	0.002
Baseline SYNTAX score	0.062	0.007
Residual Syntax Score	0.176	0.009
Procedural success	0.049	0.023

We have suggested that the standardized difference of less than 0.1 likely denote a negligible imbalance between IVUS guidance and angiograph guidance subjects.
